# ﻿*Melitapanda*, a new species of Melitidae (Crustacea, Amphipoda) from Japan

**DOI:** 10.3897/zookeys.1212.128858

**Published:** 2024-09-21

**Authors:** Ko Tomikawa, Shigeyuki Yamato, Hiroyuki Ariyama

**Affiliations:** 1 Graduate School of Humanities and Social Sciences, Hiroshima University, 1-1-1 Kagamiyama, Higashihiroshima, Hiroshima 739-8524, Japan Hiroshima University Higashihiroshima Japan; 2 Shirahama Katata 2364-64, Wakayama 649-2201, Japan Unaffiliated Wakayama Japan; 3 Osaka Museum of Natural History, Nagai Park, Higashi-Sumiyoshi, Osaka 546-0034, Japan Osaka Museum of Natural History Osaka Japan

**Keywords:** Intertidal zone, *Melitapanda*, molecular phylogeny, morphology, systematics, taxonomy, Wakayama

## Abstract

A new intertidal species of the melitid amphipod, *Melitapanda*, from the Wakayama Prefecture, Japan, is identified and described. *Melitapanda***sp. nov.** differs from the similar *M.koreana* and *M.nagatai* by its black-and-white body color, well-developed anterodistal projection of the male gnathopod 1 propodus, and telson armature. Molecular phylogenetic analyses based on the nuclear 28S rRNA and mitochondrial COI genes support that *M.panda***sp. nov.** is closely related to *M.koreana* and *M.nagatai*.

## ﻿Introduction

*Melita* Leach, 1814, encompasses melitid amphipods found in marine, brackish, and freshwater environments. It includes 63 described species worldwide (Krapp-Schickel and Sket 2015; Labay 2016; Tomikawa et al. 2018, 2022; Horton et al. 2024). Sixteen species of the genus have been recorded in Japan: *M.bingoensis* Yamato, 1987; *M.choshigawaensis* Tomikawa, Hirashima, Hirai & Uchiyama, 2018; *M.hoshinoi* Yamato, 1990; *M.koreana* Stephensen, 1944; *M.longidactyla* Hirayama, 1987; *M.miyakoensis* Tomikawa & Aoyagi, 2022 in Tomikawa et al. (2022); *M.nagatai* Yamato, 1987; *M.nunomurai* Tomikawa & Sasaki, 2022 in Tomikawa et al. (2022); *M.ogasawaraensis* Tomikawa & Sasaki, 2022 in Tomikawa et al. (2022); *M.okinawaensis* Tomikawa & Nakano, 2022 in Tomikawa et al. (2022); *M.pilopropoda* Hirayama, 1987; *M.quadridentata* Yamato, 1990; *M.rylovae* Bulyčeva, 1955; *M.setiflagella* Yamato, 1988; *M.shimizui* (Uéno, 1940); and *M.tuberculata* Nagata, 1965. However, taxonomic studies of *Melita* in the Japanese Archipelago are insufficient, and many undescribed species still remain.

In the 1990s, one of the authors (SY) found an unidentified species of *Melita* recognized by the characteristic black-and-white body coloration from the intertidal zone of Wakayama Prefecture, Japan. This species was treated as *Melita* sp. 1 by Ariyama (2022). Recently, a significant number of specimens of this species have been accumulated by the first and third authors (KT and HA). Detailed morphological analysis revealed that this species has not been previously described; therefore, it is described and named here. In recent years, molecular phylogenetic analyses have been used to elucidate the phylogenetic relationships among species of *Melita* (Tomikawa et al. 2022). The phylogenetic positions of the newly identified species in this study are also clarified.

## ﻿Material and methods

### ﻿Sampling and morphological observation

Samples of the undescribed *Melita* species were collected from intertidal waters in Wakayama Prefecture, Japan. Samples were collected by passing a hand-net over cobble and sand substrates, and the amphipods were later sorted. The samples were fixed and preserved in 99% ethanol at the site.

All appendages were dissected in 80% ethanol and mounted in a gum-chloral medium on glass slides using a stereomicroscope (Olympus SZX7). Slides were examined using a light microscope (Nikon Eclipse Ni), with appendages illustrated using a camera lucida (Nikon Y-IDT). Male gnathopod 1 and female pereopod 6 were dehydrated through a graded ethanol series, and dried using hexamethyldisilazane (HMDS) (Nation 1983). They were then sputter-coated with gold and observed using scanning electron microscopy (SEM, JSM-6510LV). Body length was measured from the rostrum tip to the telson base, along the dorsal curvature to the nearest 0.1 mm. Type specimens are deposited in the National Museum of Nature and Science, Tsukuba (**NSMT**).

### ﻿Molecular phylogeny

Genomic DNA was extracted from the appendage muscles of the samples following procedures detailed by Tomikawa et al. (2014b) and Tomikawa et al. (2014a). The primer sets for PCR and cycle sequencing reactions used in this study were as follows: 28SF and 28SR (Tomikawa et al. 2012) for 28S rRNA (28S), H3aF and H3bR (Colgan et al. 2000) for histone H3 (H3), and LCO1490 and HCO2198 (Folmer et al. 1994) for cytochrome *c* oxidase subunit I (COI). PCR and DNA sequencing were performed using the method detailed by Tomikawa (2015). The newly obtained DNA sequences were deposited in the International Nucleotide Sequence Databases (INSD) through the DNA Data Bank of Japan (**DDBJ**) (Table 1).

**Table 1.** Information and GenBank accession numbers of specimens studied in the present study. Sequences marked with an asterisk were newly obtained in this study.

Species	Voucher or isolate number	Locality	Coordinates (decimal degrees)	GenBank accession numbers		
				*COI*	*28S*	*histone H3*
*Melitashimizui*	G652	Ota River, Hiroshima, Japan	32.4050°N, 132.4371°E	LC371928	LC637781	LC637944
*Melitaokinawaensis*	NSMT-Cr 29115	Shiokawa, Motobu, Okinawa, Japan	26.6158°N, 127.8950°E	LC637583	LC637782	LC637945
*Melitachoshigawaensis*	G1391	Choshi River, Kihoku, Mie, Japan	34.1082°N, 136.2219°E	LC371923	LC637783	LC637946
*Melitanagatai*	G1392	Etajima, Hiroshima, Japan	34.1461°N, 132.4400°E	LC637584	LC637784	LC637947
*Melitasetiflagella*	G1393	Numata River, Mihara, Hiroshima, Japan	34.3889°N, 133.0528°E	LC637585	LC637785	LC637948
*Teganoshiodamari*	G1418	Unbuki Cave, Tokunoshima I., Kagoshima, Japan	27.8269°N, 128.8810°E	LC637586	LC637787	LC637950
*Melitanunomurai*	NSMT-Cr 29091	Stream near Osawa Beach, Haha-jima, Ogasawara, Japan	26.6987°N, 142.1349°E	LC637587	LC637788	LC637951
*Melitaogasawaraensis*	NSMT-Cr 29100	Stream at Hatsuneura, Chichi-jima, Ogasawara, Japan	27.0807°N, 142.2245°E	LC637591	LC637792	LC637955
***Melitapanda* sp. nov.**	NSMT-Cr 32146 (G1489)	Jogasaki, Wakayama, Japan	34.2850°N, 135.0680°E	*LC815081	*LC815084	*LC815087
***Melitapanda* sp. nov.**	NSMT-Cr 32145 (G1491)	Tagurazaki, Wakayama, Japan	34.2666°N, 135.0608°E	*LC815082	*LC815085	*LC815088
***Melitapanda* sp. nov.**	NSMT-Cr 32147 (G1492)	Jogasaki, Wakayama, Japan	34.2850°N, 135.0680°E	*LC815083	*LC815086	*LC815089
*Melitarylovae*	G1766	Shijushima I. Mukaishima, Onomichi, Hiroshima, Japan	34.3608°N, 133.1630°E	LC637597	LC637798	LC637961
*Abludomelitaklitinii*	G1767	Aidomari, Shiretoko, Hokkaido, Japan	44.1851°N, 145.3180°E	LC637598	LC637799	LC637962
*Melitakoreana*	G1769	Sasebo River, Sasebo, Nagasaki, Japan	33.1663°N, 129.7196°E	LC637600	LC637801	LC637964
*Melitahoshinoi*	G1770	Iyo-nada, Seto Inland Sea, Japan	33.4378°N, 131.8478°E	LC637601	LC637802	LC637965
*Melitamiyakoensis*	NSMT-Cr 29084	Miyahara, Hirara, Miyakojima, Okinawa, Japan	24.7830°N, 125.3372°E	LC637605	LC637806	LC637969
*Abludomelitajaponica*	G1806	Toyokunisaki, Misaki, Osaka, Japan	34.3227°N, 135.1163°E	LC637611	LC637812	LC637975
*Dulichiellatomioka*	G262	Off Tanegashima Island, Kagoshima, Japan	30.8958°N, 131.0430°E	LC637612	LC637813	LC637976
*Elasmopusnkjaf*	G1337	Miyako Island, Okinawa, Japan	24.7997°N, 125.3341°E	LC215813	LC215811	LC215815
*Gammarellacyclodactyla*	G1421	Kamikamagari Island, Kure, Hiroshima, Japan	34.1731°N, 132.7375°E	LC637613	LC637814	LC637977
*Victoriopisaryukyuensis*	G1802	Lake Manko, Naha, Okinawa, Japan	26.1936°N, 127.6816°E	LC637614	LC637815	LC637978
*Megamoeradentata*	–	Quebec, Canada	50.25°N, 66.70°W	FJ581757	–	–
*Quasimelitaformosa*	–	Quebec, Canada	50.25°N, 66.70°W	FJ581763	–	–
*Melitahergensis*	–	Minho, Litoral Norte, Viana do Castelo, Praia Norte, Portugal	41.694°N, 8.851°W	KX224061	–	–
*Melitanitida*	–	Virginia, Eastern Shore, USA	37.606°N, 75.6254°W	MH826287	–	–
*Megamoerasubtener*	–	British Columbia, Haida Gwaii, Skidegate, Canada	53.2676°N, 131.977°W	MG936114	–	–
*Megamoeradentata*	–	Washington, San Juan County, Lopez Island, USA	48.5489°N, 122.926°W	MH242844	–	–
*Exitomelitalignicola*	–	Norwegian Sea	73.5532°N, 8.282°E	JQ775392	–	–
*Exitomelitasigynae*	–	Norwegian Sea	73.5668°N, 8.1604°E	JN831764	–	–

The phylogenetic analyses were conducted based on sequences of nuclear 28S and H3, and mitochondrial COI. Three species, *Victoriopisaryukyuensis* Morino, 1991 (Eriopisidae), *Elasmopusnkjaf* Nakamura, Nakano, Ota & Tomikawa, 2019 (Maeridae), and *Gammarellacyclodactyla* (Hirayama, 1978) (Nuuanuidae), were included in the analyses as outgroup taxa. The alignment of H3 and COI was trivial, as no indels were observed. The 28S sequences were aligned using the Muscle algorithm using the software MEGA11 (Tamura et al. 2021). The lengths of the 28S, H3 and COI were found to be 1001, 331 and 658 bp, respectively. The concatenated sequences yielded 1990 bp of alignment positions.

Phylogenetic relationships were reconstructed via maximum likelihood (ML) and Bayesian inference (BI), and partitioned by 28S, H3, and the 1^st^, 2^nd^, and 3^rd^ codon positions for COI. The best evolutionary models were selected based on the corrected Bayesian information criterion (BIC) using ModelFinder (Kalyaanamoorthy et al. 2017): for 28S, TIM3+F+G4; for H3, TNe+G4; for COI, TPM3+F+G4. ML phylogenies were conducted using IQ-TREE web server (ver. 1.6.12, see http://www.iqtree.org/; Trifinopoulos et al. 2016) with 1000 ultrafast bootstrap replicates (Hoang et al. 2018) performed to estimate statistical support for branching patterns with 1000 replicates. The BI tree and Bayesian posterior probabilities were estimated using MrBayes ver. 3.2.6 (Ronquist et al. 2012), with Markov chains of 10 million generations. Parameter estimates and convergence were checked using Tracer ver. 1.7.1 (Rambaut et al. 2018), and the first 1 million trees were discarded as burn-in.

## ﻿Results

### ﻿Systematics

**Family Melitidae Bousfield, 1973**

**Genus *Melita* Leach, 1814**

#### Melita panda sp. nov.

Taxon classificationAnimaliaAmphipodaMelitidae

DE91E2B3-34D6-58C7-95F5-836FE6DB2691

https://zoobank.org/EC735281-9AAC-4DFF-BC0D-945876A8E958

**Figure 1.**
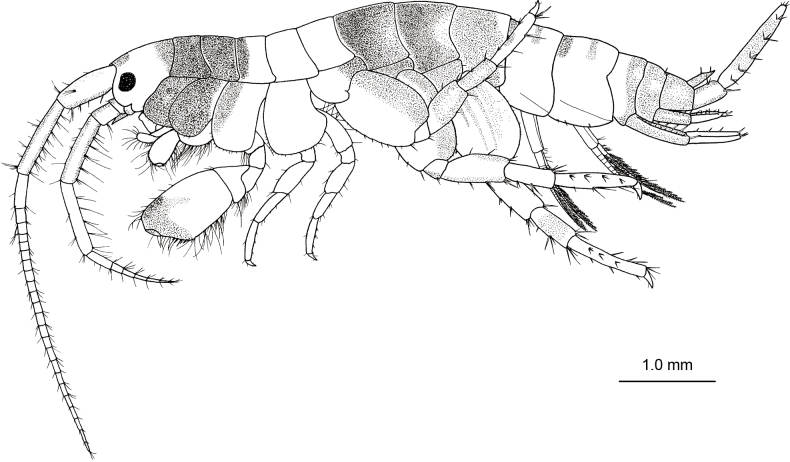
*Melitapanda* sp. nov. holotype, male 6.0 mm (NSMT-Cr 32141), habitus, lateral view.

**Figure 2.**
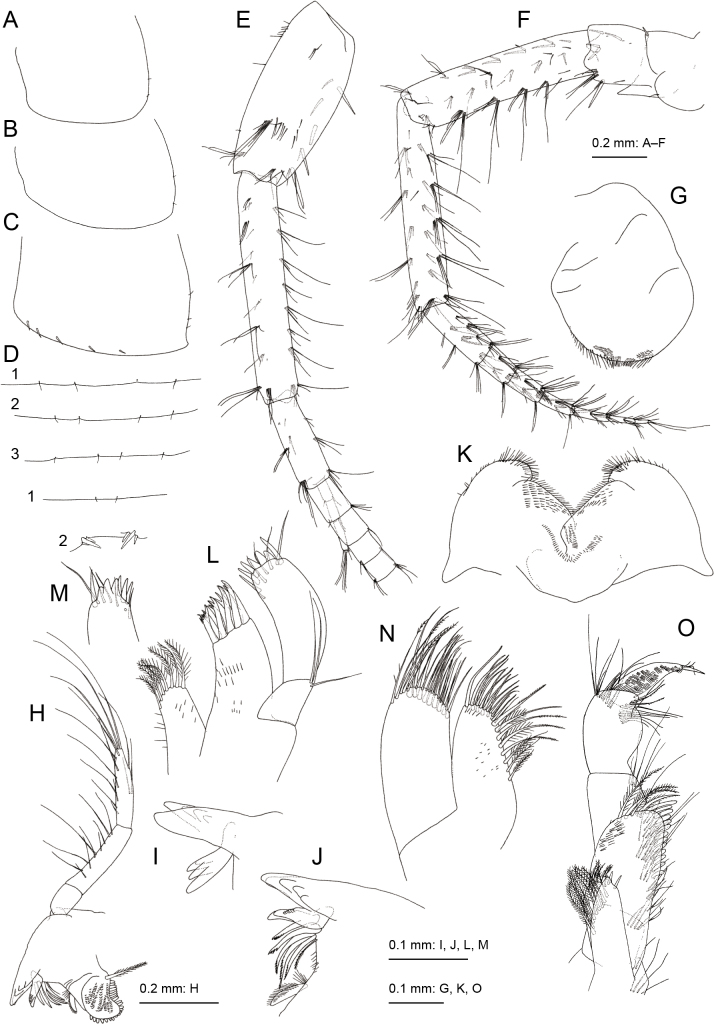
*Melitapanda* sp. nov. holotype, male 6.0 mm (NSMT-Cr 32141) **A–C** epimeral plates 1–3, lateral views **D** dorsal margins of pleonites 1–3 and urosomites 1–2, dorsal view **E** antenna 1, lateral view, some articles of main flagellum omitted **F** antenna 2, lateral view **G** upper lip, anterior view **H** right mandible, medial view **I** incisor and lacinia mobilis of left mandible, lateral view **J** incisor and lacinia mobilis of right mandible, medial view **K** lower lip, dorsal view **L** left maxilla 1, ventral view **M** apical part of right maxilla 1, ventral view **N** maxilla 2, dorsal view **O** maxilliped, dorsal view.

**Figure 3.**
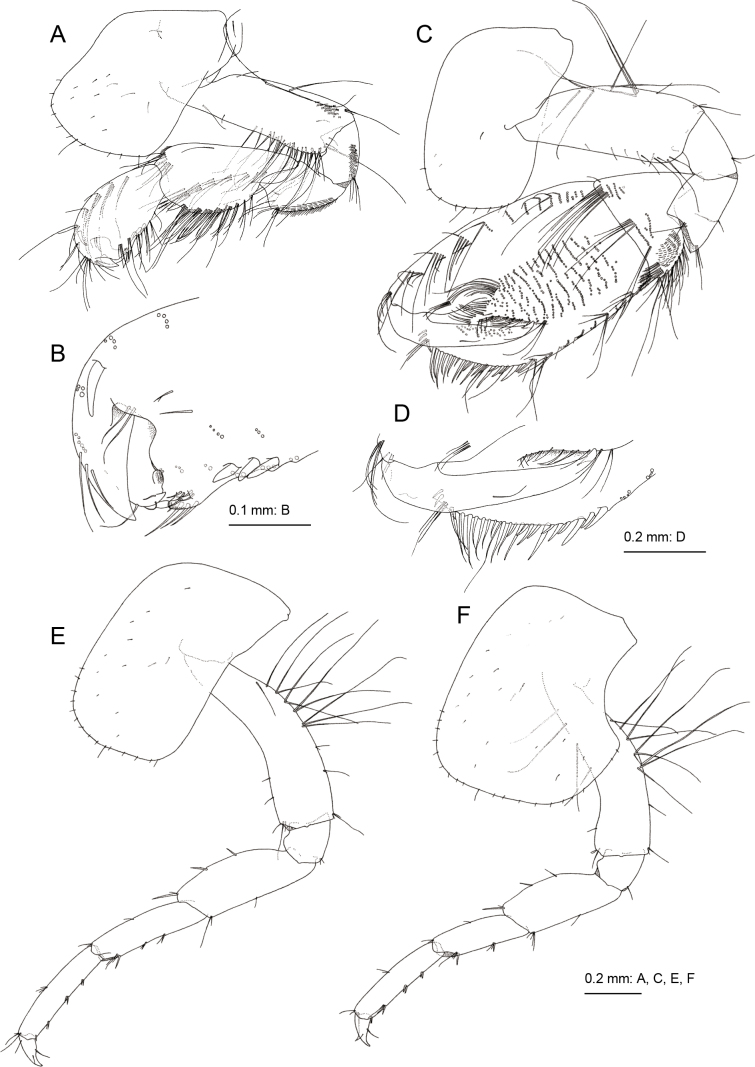
*Melitapanda* sp. nov. holotype, male 6.0 mm (NSMT-Cr 32141) **A** gnathopod 1, lateral view **B** palmar margin of propodus and dactylus of gnathopod 1, medial view **C** gnathopod 2, medial view **D** palmar margin of propodus and dactylus of gnathopod 2, medial view **E** pereopod 3, lateral view **F** pereopod 4, lateral view.

**Figure 4.**
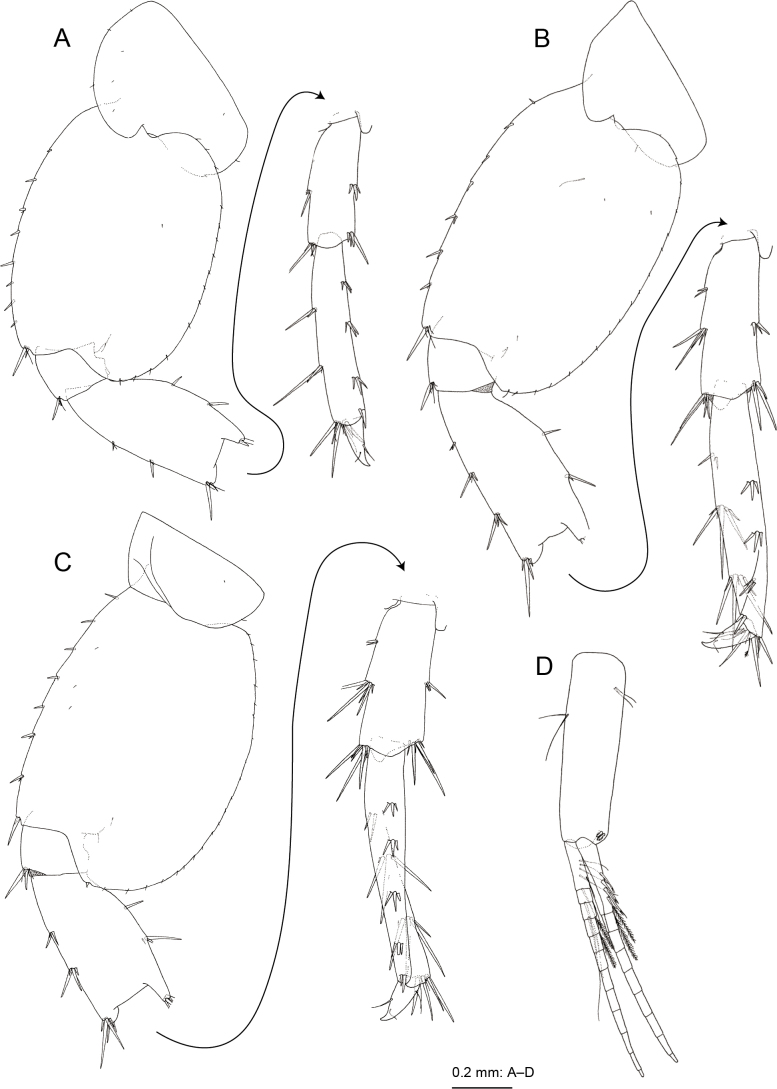
*Melitapanda* sp. nov. holotype, male 6.0 mm (NSMT-Cr 32141) **A** pereopod 5, lateral view **B** pereopod 6, lateral view **C** pereopod 7, lateral view **D** pleopod 1, medial view.

**Figure 5.**
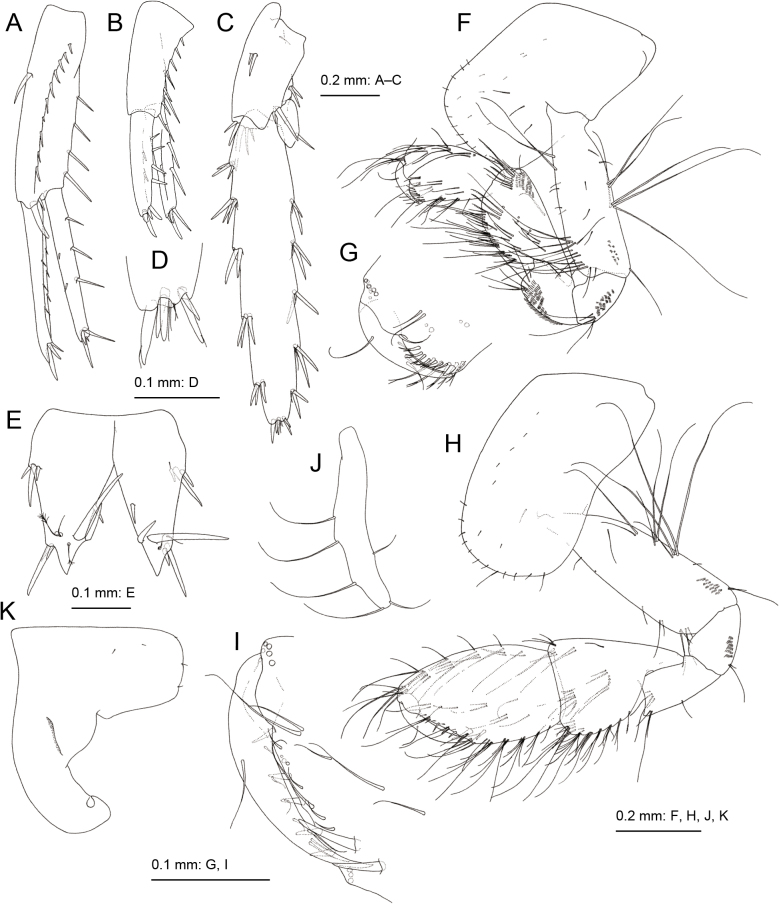
*Melitapanda* sp. nov. **A–E** holotype, male 6.0 mm (NSMT-Cr 32141) **F–K** paratype, female 4.8 mm (NSMT-Cr 32143) **A** uropod 1, dorsal view **B** uropod 2, dorsal view **C** uropod 3, dorsal view **D** uropod 3 distal part of outer ramus, dorsal view **E** telson, dorsal view **F** gnathopod 1, medial view **G** palmar margin of propodus and dactylus of gnathopod 1, medial view **H** gnathopod 2, lateral view **I** palmar margin of propodus and dactylus of gnathopod 2, medial view **J** oostegite on gnathopod 2, medial view **K** pereopod 6 coxa, lateral view.

**Figure 6.**
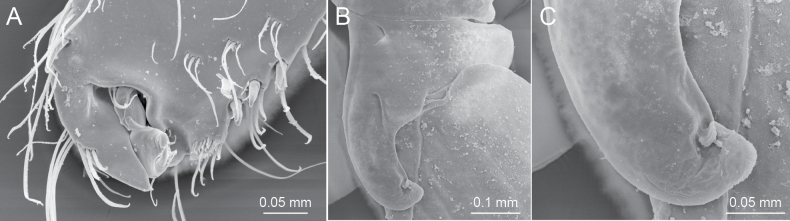
SEM photographs of *Melitapanda* sp. nov. **A** propodus and dactylus of male gnathopod 1, medial view **B** coxa of female pereopod 6, lateral view, dorsal surface partly damaged **C** distal part of coxa of female pereopod 6, lateral view. **A** paratype male 7.6 mm, NSMT-Cr 32142 **B** and **C** paratype female 4.5 mm, NSMT-Cr 32144.

Figs 1 , 2 , 3 , 4 , 5 , 6 New Japanese name: Panda-melita-yokoebi

Melita sp. 1: Ariyama, 2022: 119.

##### Material examined.

***Holotype***: • male 6.0 mm (NSMT-Cr 32141); Tagurazaki, Wakayama, Japan; (34.264603°N, 135.060835°E); collected by Hiroyuki Ariyama and Ko Tomikawa; on 25 March 2019. ***Paratypes***: • male 7.6 mm (NSMT-Cr 32142); female 4.8 mm (NSMT-Cr 32143); female 4.5 mm (SEM, NSMT-Cr 32144); data same as for the holotype • male 5.0 mm (NSMT-Cr 32145, G1491); locality same as for the holotype; collected by Hiroyuki Ariyama; on 12 July 2018 • male 5.8 mm (NSMT-Cr 32146, G1489); male 4.5 mm (NSMT-Cr 32147, G1492); Jogasaki, Wakayama, Japan; (34.2850°N, 135.0680°E); collected by Hiroyuki Ariyama; on 14 July 2018 • male 5.2 mm (NSMT-Cr 32148); female 4.3 mm (NSMT-Cr 32149); Shirahama, Wakayama, Japan; (33.691826°N, 135.336172°E); collected by Hiroki Yamada and Ko Tomikawa; on 12 November 2023.

##### Diagnosis.

Head with eyes; antennal sinus incised. Pleonites and urosomites lacking dorsal teeth. Epimeral plate 3 with weakly pointed posterodistal corner. Urosomite 2 with 3 dorsolateral robust setae on each side without distinct teeth. Antenna 1 with 4-articulate accessory flagellum. Maxilla 1 with inner plate bearing 7 plumose setae. Gnathopod 1 with basis and ischium bearing small palmate setae. Male gnathopod 1 with anterodistal projection on propodus forming rounded hood, covering almost all of dactylus, propodus with 3 and 1 robust setae on posterior margin and at the base of anterodistal projection, respectively. Male gnathopod 2 with subquadrate propodus setaceous on medial surface, angle between posterior and palmar margins of propodus being approximately 140°. Female coxa 6 hooked with anterior lobe 1.2 times deeper than width of coxa. Uropod 3 with 1-articulate outer ramus. Telson longer than its width.

##### Description.

**Holotype, male, NSMT-Cr 32141.** Head (Fig. 1) almost as long as pereonites 1 and 2 combined; rostrum short; eyes developed, oval; lateral cephalic lobe rounded; antennal sinus incised. Pereonites 1–7 (Fig. 1) dorsally smooth. Epimeral plates 1–3 (Fig. 2A–C) with 2, 2 and 3 short setae on posterior margin; epimeral plate 3 posterodistal corner weakly produced, ventral margin with 5 robust setae. Pleonites 1–3 dorsal margins without teeth, each with 4 short setae and that of urosomite 1 with 2 short setae (Fig. 2D); dorsal margin of urosomite 2 with 3 robust setae on each side (Fig. 2D).

Antenna 1 (Fig. 2E) length 0.7 times that of body; length ratio of peduncular articles 1–3 as 1.0: 1.3: 0.5; ventral margin of peduncular article 1 with 3 robust setae, posterodistal corner with robust seta; primary flagellum 23-articulate with a few setae; accessory flagellum 4-articulare, terminal article short. Antenna 2 (Fig. 2F) length 0.6 times that of antenna 1; peduncular article 2 with robust seta on anterodistal corner; peduncular article 3 with robust setae on medial surface and posterodistal corner; peduncular articles 4 and 5 with 4 clusters of setae on posterior margins, peduncular article 5 1.1 times as long as article 4; flagellum 8-articulate, article 1 2.3 times as long as article 2; calceoli absent.

Upper lip (Fig. 2G) with convex, rounded ventral margin bearing minute setae. Left and right mandible with 5-dentate incisor (Fig. 2H–J); left lacinia mobilis (Fig. 2I) 4-dentate, right lacinia mobilis (Fig. 2J) with 2 large and several small teeth; left and right accessory setal rows each with five-bladed setae and plumose seta; molar process triturative with plumose seta; palp tri-articulate, length ratio of articles 1–3 1.0: 2.3: 2.3, article 1 marginally bare, article 2 with 10 setae on ventral margin, article 3 with 12 and 3 setae on ventral and dorsal margins, respectively. Lower lip (Fig. 2K) with broad outer lobes bearing minute setae, mandibular lobes narrow; inner lobes indistinct. Maxilla 1 (Fig. 2L, M) with inner plate bearing 7 plumose setae; outer plate rectangular with 9 serrate robust setae; palp 2-articulate; article 1 with 3 long setae on laterodistal corner; article 2 arched, outer margin bare, apical margin with robust and slender setae. Maxilla 2 (Fig. 2N) with inner plate lacking oblique inner row of setae; outer plate slightly longer than inner plate. Maxilliped (Fig. 2O) with inner plate not reaching half of palp article 2, bearing 3 robust setae distally; outer plate ovate, exceeding half of palp article 2, apical margin with plumose setae, inner submargin with robust setae; palp 4-articulate, medial margin of article 2 lined with setae, article 3 weakly expanded medially, article 4 with fine facial setae and nail.

Gnathopod 1 (Fig. 3A, B) smaller than gnathopod 2; coxa weakly expanded ventrally, ventral margin with setae; basis, anterior and posterior margins with long setae, posterodistal submargin with tiny palmate setae; ischium with tiny palmate setae; merus ventral margin with small setae; carpus not lobate, length 1.2 times that of propodus, anterodistal corner with palmate setae, posterior margin with clusters of setae; propodus 1.9 times as long as wide, anterodistal projection forming hood, covering almost all of dactylus (Figs 3B, 6A), robust seta at the base of anterodistal projection, posterior margin with 3 robust setae, palmar margin with slender setae; dactylus short, not exceeding palmar margin. Gnathopod 2 (Fig. 3C, D) with subrectangular coxa, bearing setae on ventral margin; basis with long setae at anterodistal corner and on posterior margin, posterodistal submargin without palmate setae; ischium without palmate setae; carpus not lobate, length 0.4 times of that of propodus; propodus large, subquadrate, length 1.5 times as long as wide, angle between posterior and palmar margins being approximately 140°, medial surface setaceous, palmar margin oblique with robust setae becoming thinner toward end; dactylus not exceeding palmar margin.

Pereopod 3 (Fig. 3E) with subrectangular coxa, ventral margin with setae; basis arched, anterior margin with short setae, posterior margin with long and short setae; length ratio of merus, carpus, propodus and dactylus 1.0: 0.9: 0.9: 0.3; merus with robust setae on anterior margin, carpus and propodus with robust setae on posterior margins. Pereopod 4 (Fig. 3F) with expanded coxa bearing posterior concavity, with ventral setae; basis weakly arched, anterior and posterior margins with long and short setae; length ratio of merus, carpus, propodus and dactylus 1.0: 0.8: 0.9: 0.3; carpus and propodus with robust setae on posterior margins. Pereopod 5 (Fig. 4A) with bilobate coxa, anterior lobe large with small seta on anterior margin, posterior lobe with small setae on posterior margin; basis ovate with posterodistal lobe, anterior margin with robust setae, posterior margin with short setae; merus weakly expanded, length 2.2 times as long as wide. Pereopod 6 (Fig. 4B) with bilobate coxa, anterior lobe marginally bare, posterior lobe with small seta on ventral margin and posterodistal corner; basis slender, ovate, anterior margin with robust setae, posterior margin with short setae, posterodistal corner lobate; merus length 2.4 times as long as wide. Pereopod 7 (Fig. 4C) with coxa, bearing seta on posterior margin; basis subovate, anterior margin with robust setae, posterior margin weakly serrate with short setae, posterodistal corner lobate; merus length 2.1 times as long as wide.

Coxal gills present on gnathopod 2, and pereopods 3–6.

Pleopods 1–3 (Fig. 4D) peduncles with paired retinacula on inner distal margin and facial setae; inner ramus with bifid plumose setae (clothes-pin setae) on inner basal margin.

Uropod 1 (Fig. 5A) extending beyond uropod 2; peduncle length 1.3 times longer than inner ramus, with basofacial robust seta; inner ramus almost as long as outer ramus, with 3 inner and 2 outer marginal robust setae; outer ramus with 3 robust setae on inner and outer margins, respectively. Uropod 2 (Fig. 5B) not extending beyond peduncle of uropod 3; peduncle length 1.1 times longer than inner ramus; inner ramus almost as long as outer ramus, with 2 and 1 robust setae on inner and outer margins, respectively; outer ramus with 2 inner and 4 outer marginal robust setae. Uropod 3 (Fig. 5C) with peduncle not extending beyond telson, 0.4 times as long as outer ramus; inner ramus length 0.2 times that of outer ramus, with subdistal robust seta; outer ramus with single article, straight, length 5.4 times that of outer ramus width, inner and outer margins each with 5 clusters of robust setae, distal part with robust and slender setae, longest distal slender seta shorter than longest distal robust seta.

Telson (Fig. 5E) length 1.1 times longer than wide, almost completely cleft, each lobe with 1 medial, 4–5 subdistal and 3 lateral robust setae.

**Female (paratype, NSMT-Cr 32143), sexually dimorphic characters.** Gnathopod 1 (Fig. 5F, G) with carpus length 1.2 times that of propodus; propodus palmar margin vertical, with 2 rows of slender setae, palmar angle with robust seta. Gnathopod 2 (Fig. 5H, I) with elongate coxa; carpus length 0.8 times that of propodus; propodus 1.7 times as long as wide, palmar margin oblique, with 4 robust setae along palmar margin and 2 robust setae on palmar corner.

Coxa of pereopod 6 (Figs 5K, 6B, C) with anterior lobe 1.2 times deeper than width of coxa, strongly hooked, bearing slit-like shallow “pocket” at base, trailing edge near tip of anterior lobe loosely turned up.

Oostegites (Fig. 5J) present on gnathopod 2 and pereopods 3–5, narrow with setae.

##### Coloration in life.

Black pattern on white background. The black pattern is found in the following areas: antennae 1 and 2 peduncular articles; part of head, pereonites 1, 2, 3 (part), 5–7; coxae 1, 2, 3 (part), 5 (part), 6, 7; distal part of gnathopod 2 propodus; posterior half of bases of pereopods 5–7; dorsal part of pleonites 1 and 2, urosomite 1 posterior half, urosomites 2 and 3; and uropods 1 and 2 peduncles, uropod 3. Coloration is almost identical between males and females, but females lack the black area on gnathopod 2. Colors remain largely unchanged after ethanol fixation.

##### Etymology.

The species name “panda” is derived from its black-and-white body coloration, which resembles that of the giant panda *Ailuropodamelanoleuca*.

##### Molecular phylogeny.

**Figure 7.**
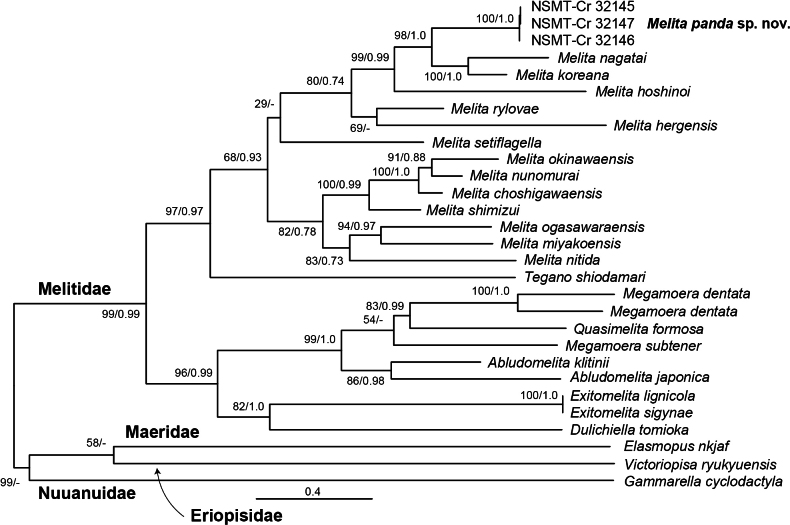
Maximum likelihood tree for 1990 bp of nuclear 28S rRNA, histone H3, and mitochondrial cytochrome *c* oxidase subunit I markers. Numbers at nodes represent ultrafast bootstrap values for maximum likelihood and Bayesian posterior probabilities.

The obtained ML tree exhibited a topology almost identical to that of the BI tree (Fig. 7). Melitidae formed a monophyletic group with high statistical support (USB = 99%, BPP = 0.99). The monophyly of *Melita* was supported by the BI tree (BPP = 0.93) but less so by the ML tree (USB = 0.68). *Melitapanda* sp. nov. was the sister taxon of *M.nagatai* and *M.koreana* among the 14 species of *Melita* used in the analyses (USB = 98%, BPP = 1.0). These three species formed a monophyletic group with *M.hoshinoi* (USB = 99%; BPP = 0.99).

## ﻿Discussion

In the genus *Melita*, 29 species with pleonites and urosomites lacking dorsal teeth, urosomite 2 bearing robust dorsal setae, and uropod 3 bearing a single-article outer ramus have been recorded (Momtazi et al. 2014; Krapp-Schickel and Sket 2015; Tomikawa et al. 2018, 2022). *Melitapanda* sp. nov. is easily distinguished from these species by the anterodistal projection of the male gnathopod 1 propodus forming a rounded hood that almost completely covers the dactylus.

*Melitapanda* sp. nov. is morphologically similar to *M.koreana* and *M.nagatai* in sharing features such as urosomite 2 with three dorsal robust setae on each side, a head with eyes and incised antennal sinus, maxilla 1 with seven setae on the inner plate and setae on palp article 1, a male gnathopod 1 propodus with a developed anterodistal projection, a male gnathopod 2 with a setaceous subrectangular propodus, pereopods 3 and 4 with short dactylus, and a uropod 3 outer ramus that is 1-articulate. However, *M.panda* sp. nov. differs from *M.koreana* and *M.nagatai* in several distinct features. Compared to *M.koreana*, *M.panda* sp. nov. has black-and-white body color (*M.koreana* is almost all gray), an anterodistal projection of the male gnathopod 1 propodus covering dactylus (*M.koreana* does not completely cover it), male gnathopod 1 propodus with three and one robust setae on posterior margin and at the base of anterodistal projection, respectively (*M.koreana* lacks these setae but has a robust seta on palmar corner), and a telson with lateral setae (*M.koreana* lacks these setae). Compared to *M.nagatai*, *M.panda* sp. nov. has a black-and-white body color (*M.nagatai* is almost all gray), lacks setae at the base of maxilla 1 palp article 1 (*M.nagatai* has setae present), has an anterodistal projection of the male gnathopod 1 propodus that covers the dactylus (*M.nagatai*’s projection does not completely cover it), male gnathopod 1 propodus with three and one robust setae on posterior margin and at the base of anterodistal projection, respectively (*M.nagatai* lacks these setae but has a robust seta on palmar corner), and has a telson with lateral setae (*M.nagatai* lacks these setae). The close relationship between *M.panda* sp. nov. and *M.koreana* + *M.nagatai* was also supported by the molecular phylogenetic analyses (Fig. 7).

*Melitapanda* sp. nov. shares similarities with *M.bingoensis* in the shape of mouthparts, male gnathopods 1 and 2, and female pereopod 6 with a slit-like shallow “pocket” at the coxa base. Nonetheless, it differs from *M.bingoensis* in the following aspects (with *M.bingoensis* features in parentheses): urosomite 2 with three (*M.bingoensis* has two) robust setae on each side, anterodistal projection of male gnathopod 1 propodus covering the dactylus (while in *M.bingoensis*, it does not completely cover it), coxa of female pereopod 6 deeper than wide (in *M.bingoensis*, it is wider than deep), and telson with lateral setae (which is absent in *M.bingoensis*). Although, *M.bingoensis* was not included in our molecular phylogenetic analyses, based on morphological similarities, *M.panda* sp. nov. is also presumed to be phylogenetically close to *M.bingoensis*.

### ﻿Key to species of *Melita* from Japan

**Table:** 

1	Uropod 1 outer ramus 1-articulate	**2**
–	Uropod 1 outer ramus 2-articulate	**14**
2	Pleonites 1–3 each with dorsal teeth	***M.tuberculata* Nagata, 1965**
–	Pleonites 1–3 without dorsal teeth	**3**
3	Eyes absent	***M.miyakoensis* Tomikawa & Aoyagi, 2022**
–	Eyes present	**4**
4	Antennal sinus of head absent; pereopods 3 and 4 with long, feeble dactyli	***M.longidactyla* Hirayama, 1987**
–	Antennal sinus of head present; pereopods 3 and 4 with short, stout dactyli	**5**
5	Antennal sinus of head incised	**6**
–	Antennal sinus of head rounded or right-angled	**10**
6	Antenna 2 flagellum densely setose	***M.setiflagella* Yamato, 1988**
–	Antenna 2 flagellum sparsely setose	**7**
7	Black-and-white body color; anterodistal projection of male gnathopod 1 propodus fully covering dactylus	***M.panda* sp. nov.**
–	Almost all gray body color; anterodistal projection of male gnathopod 1 propodus not completely covering dactylus	**8**
8	Urosomite 2 without dorsal teeth	***M.koreana* Stephensen, 1944**
–	Urosomite 2 with a minute dorsal tooth on each side	**9**
9	Male gnathopod 1 propodus with robust seta on palmar corner, lacking robust setae on medial surface; female coxa 6 deeper than wide	***M.nagatai* Yamato, 1987**
–	Male gnathopod 1 propodus without robust seta on palmar corner, bearing robust setae on medial surface; female coxa 6 shallower than wide	***M.bingoensis* Yamato, 1987**
10	Telson shorter than wide	***M.nunomurai* Tomikawa & Sasaki, 2022**
–	Telson as long as or longer than wide	**11**
11	Male gnathopod 1 propodus with anterodistal hood; female coxa 6 deeper than wide; female coxae 1 and 2 with concavity in anterior edge bearing numerous setae	***M.ogasawaraensis* Tomikawa & Sasaki, 2022**
–	Male gnathopod 1 propodus without anterodistal hood; female coxa 6 wider than deep; female coxae 1 and 2 without concavity in anterior edge	**12**
12	Male uropod 3 outer ramus approximately 5 times width; female coxa 6 wider than deep	***M.shimizui* (Uéno, 1940)**
–	Male uropod 3 outer ramus longer than 7 times width; female coxa 6 as wide as depth	**13**
13	Male gnathopod 2 propodus elevated proximally; female gnathopod 2 basis with small palmate setae	***M.okinawaensis* Tomikawa & Nakano, 2022**
–	Male gnathopod 2 propodus not elevated proximally; female gnathopod 2 basis without small palmate setae	***M.choshigawaensis* Tomikawa, Hirashima, Hirai & Uchiyama, 2018**
14	Uropod 3 outer ramus with long setae	***M.quadridentata* Yamato, 1990**
–	Uropod 3 outer ramus without long setae	**15**
15	Maxilla 1 palp article 1 without setae; male gnathopod 2 propodus with squarish palmar corner	***M.rylovae* Bulyčeva, 1955**
–	Maxilla 1 palp article 1 with setae; male gnathopod 2 propodus with transverse palmar margin	**16**
16	Setae on maxilla 1 palp article 1 reaching distal part of palp article 2; pereopods 5–7 with basis not extremely expanded	***M.hoshinoi* Yamato, 1990**
–	Setae on maxilla 1 palp article 1 not reaching distal part of palp article 2; pereopods 5–7 with basis extremely expanded	***M.pilopropoda* Hirayama, 1987**

## ﻿Conclusion

*Melitapanda* sp. nov. is clearly distinguished from its congeners by morphological and molecular features, indicating that it is an undescribed distinct species. The discovery of this new species sheds light on the high species diversity of *Melita* in the waters around Japan. Molecular phylogenetic analyses were shown to be useful for inferring phylogenetic relationships among species of the genus and detecting undescribed species.

## Supplementary Material

XML Treatment for Melita panda

## ﻿Additional information

### Author ORCIDs

Ko Tomikawa https://orcid.org/0000-0003-1521-9016

Hiroyuki Ariyama https://orcid.org/0000-0001-7312-5187

### Data availability

All of the data that support the findings of this study are available in the main text.
